# Biodistribution and efficacy of an anti-TENB2 antibody-drug conjugate in a patient-derived model of prostate cancer

**DOI:** 10.18632/oncotarget.27263

**Published:** 2019-10-22

**Authors:** C. Andrew Boswell, Daniela Bumbaca Yadav, Eduardo E. Mundo, Shang-Fan Yu, Jennifer Arca Lacap, Aimee Fourie-O’Donohue, Katherine R. Kozak, Gregory Z. Ferl, Crystal Zhang, Jason Ho, Sheila Ulufatu, Leslie A. Khawli, Kedan Lin

**Affiliations:** ^1^Genentech Research and Early Development, South San Francisco, 94080 CA, USA; ^2^Present address: Department of Safety Assessment, Nektar Therapeutics, San Francisco, 94158 CA, USA; ^3^Present address: Department of Pathology and Laboratory Medicine, Keck School of Medicine of USC, Los Angeles, 90033 CA, USA; ^4^Present address: Clinical Development and US Operation, Innovent Biologics, South San Francisco, 94080 CA, USA

**Keywords:** antibody-drug conjugate, prostate cancer, pharmacokinetics, TENB2, imaging

## Abstract

TENB2, a transmembrane proteoglycan protein, is a promising target for antibody drug conjugate (ADC) therapy due to overexpression in human prostate tumors and rapid internalization. We previously characterized how predosing with parental anti-TENB2 monoclonal antibody (mAb) at 1 mg/kg in a patient-derived LuCap77 explant model with high (3+) TENB2 expression could (i) block target-mediated intestinal uptake of tracer (& 0.1 mg/kg) levels of radiolabeled anti-TENB2-monomethyl auristatin E ADC while preserving tumor uptake, and (ii) maintain efficacy relative to ADC alone. Here, we systematically revisit this strategy to evaluate the effects of predosing on tumor uptake and efficacy in LuCap96.1, a low TENB2-expressing (1+) patient-derived model that is more responsive to ADC therapy than LuCap77. Importantly, rather than using tracer (& 0.1 mg/kg) levels, radiolabeled ADC tumor uptake was assessed at 1 mg/kg – one of the doses evaluated in the tumor growth inhibition study – in an effort to bridge tissue distribution (PK) with efficacy (PD). Predosing with mAb up to 1 mg/kg had no effect on efficacy. These findings warrant further investigations to determine whether predosing prior to ADC therapy might improve therapeutic index by preventing ADC disposition and possible toxicological liabilities in antigen-expressing healthy tissues.

## INTRODUCTION

With four approvals and another ~80 in clinical development, antibody-drug conjugates (ADCs) represent an important class of therapeutics [1, 2]. The intent of ADC therapy is to combine the antigen-specificity of antibodies with the potency of cytotoxic drugs [3, 4]. However, aside from the approval of Kadcyla™ (ado-trastuzumab emtansine) for the treatment of HER2-positive metastatic breast cancer [5], success in ADC therapy has largely been confined to hematologic cancers, prompting the continued pursuit of strategies to improve tumor specificity, and minimize normal tissue toxicity. The ability of ADC therapeutics to achieve desirable therapeutic indices hinges on the balance between specific delivery of toxic chemotherapeutics to tumors and minimizing the risk of side effects to normal tissues [6].

TENB2, also known as tomoregulin or transmembrane protein with epidermal growth factor (EGF)-like and two follistatin-like domains (TMEFF), is a proteoglycan over-expressed in human prostate tumors [7], and has been pursued as an ADC target [8, 9]. Thio-anti-TENB2-MC-vc-PAB-MMAE is a humanized anti-TENB2 ThioMab [10] conjugated to a potent anti-mitotic auristatin drug, monomethyl auristatin E (MMAE) through a maleimido-caproyl-valine-citrulline-para-amino-benzyloxy carbonyl (MC-vc-PAB) linker designed to be cleaved by lysosomal proteases [11].

Predosing with a carefully chosen dose of an unconjugated parental monoclonal antibody (mAb) is a potential means of decreasing ADC uptake by saturating receptors expressed in non-tumor tissues while preserving tumor uptake. The predosing strategy relies on blockage of binding sites with a less potent unconjugated antibody [12], and has been successfully applied in nuclear imaging and radioimmunotherapy [13–15]. Although predosing has been successful in increasing the tumor localization of radioimmunoconjugates in xenograft models and in the clinic [16–18], its effects on ADC efficacy are less understood.

For our previous investigations on predosing for ADCs, we chose the LuCaP77 tumor explant model [19] based on high tumor expression of hTENB2 together with additional mTENB2 expression in murine intestine [20]. We observed that the affinity of our anti-TENB2 ADC (and parental mAb) is single digit nanomolar and comparable against both human and mouse TENB2 [21]. We also reported that predosing with parental anti-TENB2 mAb resulted in reduced target-mediated drug distribution (TMDD) of a tracer (& 0.1 mg/kg) dose of radiolabeled ADC in mouse intestines (low expressing) without affecting uptake in LuCap77 tumor explants (high expressing, 3+) [20]. Despite impressive tumor uptake, only a modest pharmacodynamic (PD) response to the ADC was observed, possibly due to drug resistance or other mechanisms [9]. For instance, the discrepancy in sensitivity to MMAE may be related to recently reported differences in phenotypes between these two models [22].

**Figure 1:**
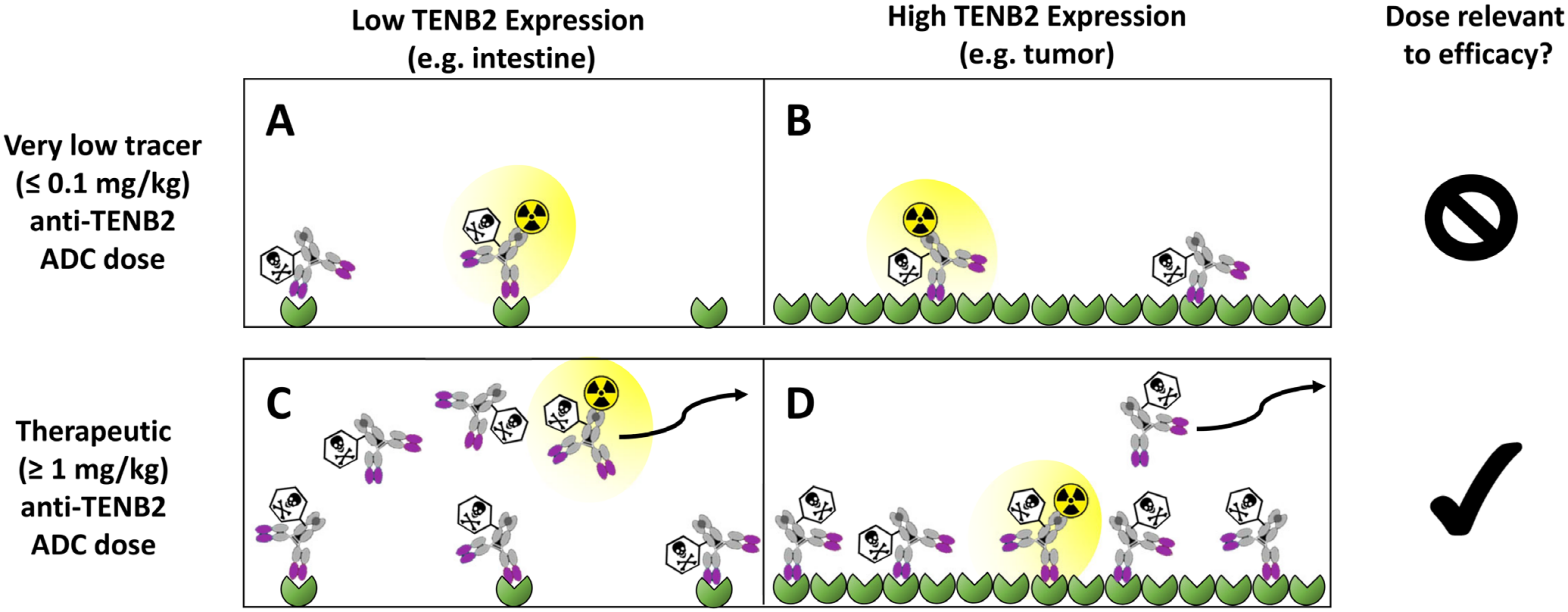
Conceptual depiction of TENB2 receptor occupancy by anti-TENB2 ADC molecules, in the absence of antibody predosing, in low TENB2 expressing tissues (e.g. murine intestine) (**A, C**) and high TENB2 expressing tissues (e.g. tumor) (**B, D**). A very low ‘tracer’ dose (≤ 0.1 mg/kg) was employed in our previous studies to focus more on the intestinal antigen sink (A–B). In contrast, the present manuscript utilizes higher doses (≥ 1 mg/kg) that are relevant to efficacy (C–D). Three quantities must be considered when interpreting these tissue uptake values: 1) target concentration, which is low in intestine (A, C) and high in tumor (B, D), 2) absolute drug dose, which was low in the first study (A, B) and high in the current study (C, D), and 3) specific radioactivity of the administered drug solution, which, since total radioactivity is fixed across all studies to ensure appropriate gamma counting detection efficiency, was *high* in the first study (A, B) and *low* in the current study (C, D). As depicted qualitatively in panels A–D, these combinations of tissue-specific target concentrations, absolute drug doses and specific radioactivities across our studies resulted in unlabeled drug outcompeting radiolabeled drug for TENB2 binding in intestine but not in tumor when increasing total drug dose from tracer to therapeutic levels. Curved arrows indicate that unbound ADC molecules may exit the interstitial space and return to systemic circulation via lymphatic drainage.

Predosing with unconjugated anti-TENB2 mAb in the LuCaP77 tumor explant model previously yielded a differential effect on ADC in that it reduced non-tumor uptake and target-mediated clearance but did not alter tumor growth inhibition relative to ADC administration without predosing [20]. These studies confirmed our hypothesis that predosing at a suitable dose level would saturate the low to moderate peripheral antigen expression while maintaining tumor uptake. However, the extremely high TENB2 expression in LuCaP77 explants relative to intestines could be considered as a low bar, prompting us to pursue tumor models with lower TENB2 expression. An additional concern was the very limited dose response, especially at the lower dose level, of the LuCaP77 model to ADC therapy [20]. To address these limitations and build on our previous knowledge, we have conducted a new series of biodistribution and efficacy studies using LuCaP96.1, a tumor model having lower TENB2 expression than LuCap77, but with clear dose dependency to anti-TENB2-MMAE ADC therapy [9]. Furthermore, in our previous biodistribution studies, we deliberately employed very low (& 0.1 mg/kg) tracer doses, far lower than those used in efficacy studies (0.75 and 2.5 mg/kg), in order to assess the interaction of the ADC with the low level antigen sink in normal tissues. However, the present studies were performed at no less than 1 mg/kg, enough to saturate the gut antigen sink with the additional unlabeled drug added to the dosing solution (thus blocking intestinal uptake of radiolabeled ADC, see Figure 1), in order to bridge the distribution and efficacy studies so that the consequences of predosing on tumor pharmacokinetics and pharmacodynamics may be directly related.

## RESULTS

### PK modeling

**Figure 2:**
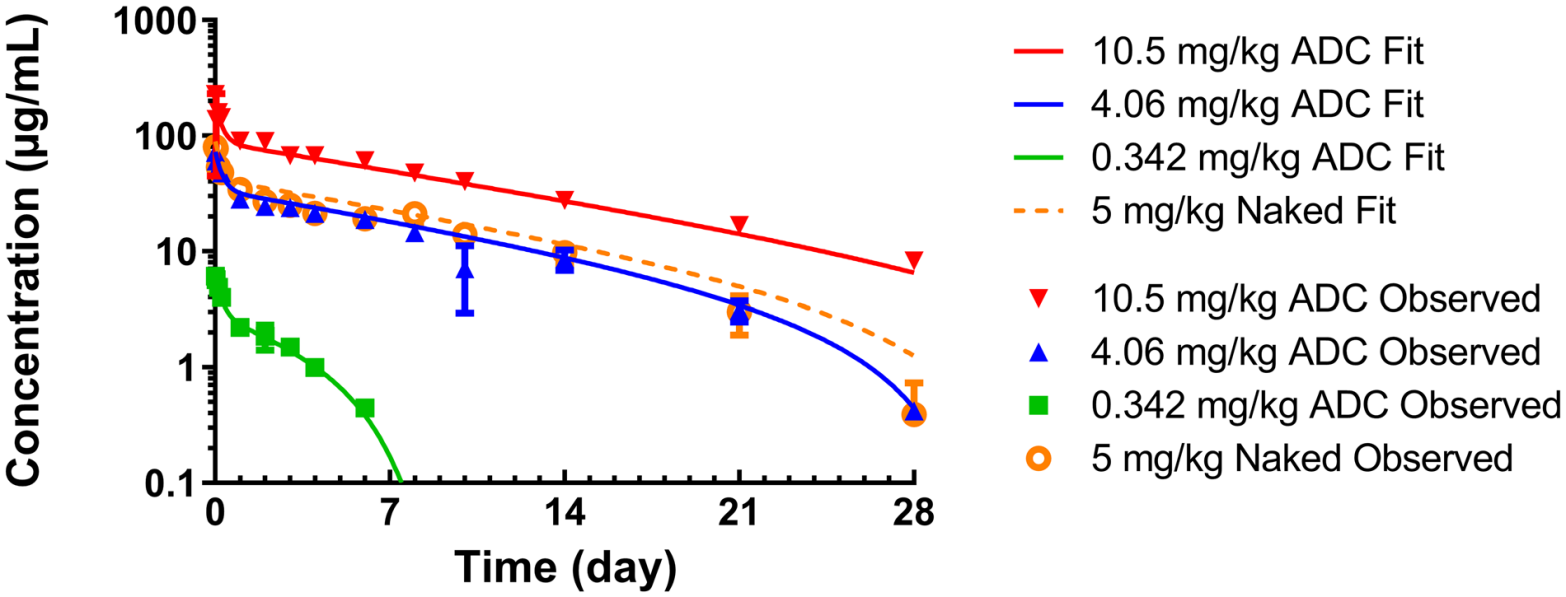
gPKPDSim [23] was used to fit a two-compartment TMDD model to previously published PK data for anti-TENB2 ADC in normal mice [21] for parameter estimation. The symbols represent the observed data, while the lines represent the model fit.

gPKPDSim [23] was used to fit a two-compartment model with non-linear clearance (Vm, Km) to previously published PK data for anti-TENB2 ADC [21] for parameter estimation (Figure 2). The parameter values (± estimation error) estimated from PK data of ADC at doses ranging from 0.342 to 10.5 mg/kg were 55.5 ± 0.990 mL/kg for V1, 58.6 ± 3.3 mL/kg for V2, 8.97 ± 0.477 mL/kg/day for CL, 105 ± 24.3 mL/kg/day for Cld, 38.1 ± 3.44 μg/day/kg for Vm, and 0.142 ± 0.0960 μg/mL for Km.

### ADC biodistribution/PK and imaging study

**Figure 3:**
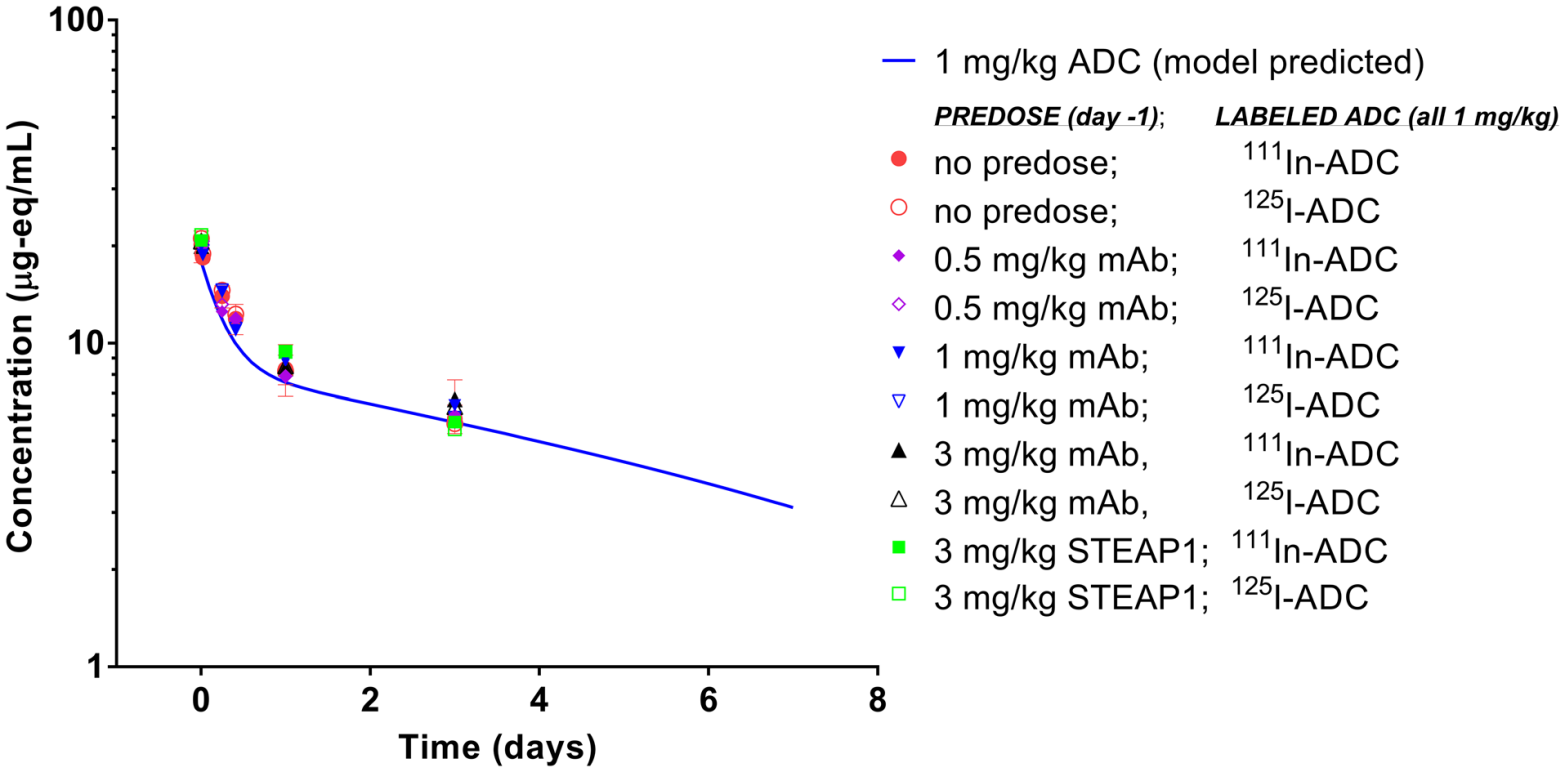
Blood pharmacokinetics of anti-TENB2 ADC (1 mg/kg) labeled with ^125^I and ^111^In in LuCaP96.1 tumor explant-bearing mice. Observed data points, expressed as microgram equivalents per mL of plasma, are in agreement with the simulated PK curve for ADC at 1 mg/kg as well as across various ADC dose levels, predose levels, and with both radiolabels. Axis ranges are intentionally expanded to allow direct comparison to the sparse PK data from mice in the efficacy study (Figure 6) whose simulated PK curve at 1 mg/kg is identical.

To assess whether antigen occupancy by unconjugated antibody can modulate PK exposure and/or impact the distribution of ADC between tumor and normal tissue, we predosed the tumor bearing mice with escalating doses of anti-TENB2 antibody, and monitored uptake of ^111^In-ADC in blood, tumor, and selected tissues. Predosing with anti-TENB2 had little to no effect on blood PK (Figure 3), suggesting that the chosen ADC dose of 1 mg/kg was large enough to saturate the TENB2 expressed in murine intestine during the first three days after dosing (see Figure 1), in contrast with the previously observed nonlinear clearance following a very low (& 0.1 mg/kg) tracer dose of the same radiolabeled ADC in both normal [21] and tumor-bearing [20] mice. For instance, blood concentrations of ^125^I-ADC at 24 h were 20 ± 2, 18 ± 2, and 20 ± 4%ID/mL with 0, 0.5, and 1 mg/kg predose, respectively, with very similar data for the ^111^In-labeled ADC. By 72 h, these concentrations had decreased to 12 ± 1, 13 ± 2, and 12.5 ± 0.8%ID/mL, respectively, and the corresponding values for mice receiving a 3 mg/kg predose were 13 ± 1 for anti-TENB2 and 11 ± 3 for anti-STEAP1 (a non-competing control antibody). All observed radioactive blood PK data agreed quite well with the simulated PK curve for 1 mg/kg ADC (Figure 3).

**Figure 4:**
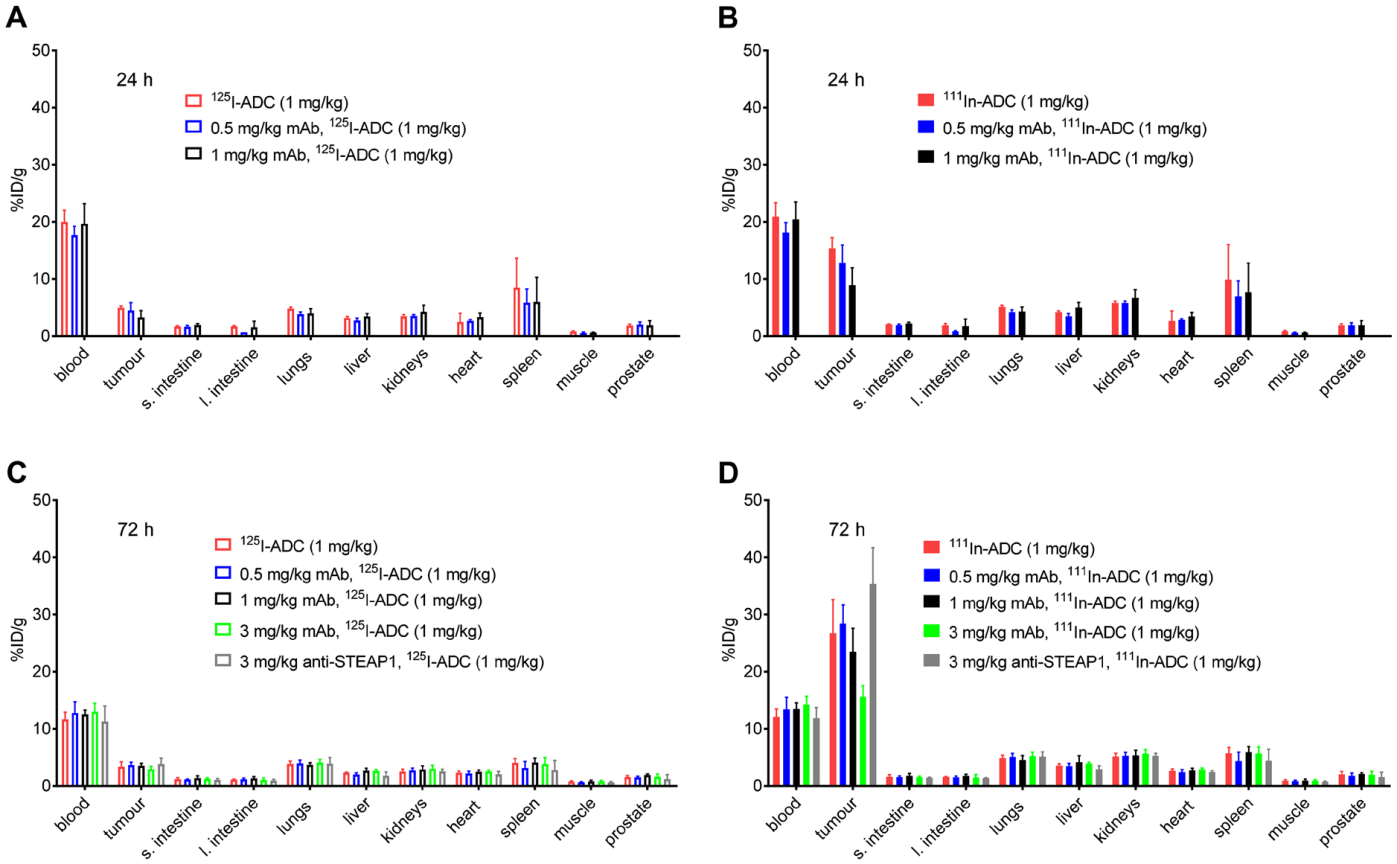
Tissue distribution at 24 h post-injection of ^125^I-ADC (**A**) and ^111^In-ADC (**B**) at 24 hours, and at 72 h post-injection of ^125^I-ADC (**C**) and ^111^In-ADC (**D**). A single dose of anti-TENB2 ADC was fixed at 1 mg/kg, preceded 24 h prior by an anti-TENB2 mAb predose of 0, 0.5, or 1 mg/kg. Tissue uptake of radiolabeled ADC is expressed as percentage of injected dose per gram of tissue (%ID/g).

Overall, predosing with anti-TENB2 had little to no effect on tissue distribution, with the exception of tumor, for which there was a trend towards dose-dependent reduction in uptake, especially at the 3 mg/kg predose level (Figure 4). At 24 h, little to no effects of predosing were detected in any normal tissue whether using non-residualizing ^125^I (Figure 4A) or residualizing ^111^In (Figure 4B) as a probe for anti-TENB2 uptake. Tumor uptakes of ^125^I-ADC at 24 h were 4.9 ± 0.3, 5 ± 1, and 3 ± 1%ID/g with 0, 0.5, and 1 mg/kg predose, respectively. Cell internalization of the ADC was evident, as the respective values for ^111^In-ADC were much higher at 15 ± 2, 13 ± 3, and 9 ± 3%ID/g. Uptake of ^125^I-ADC in other tissues showed no dose dependence and ranged from 6–8%ID/g in spleen to 0.6–0.8%ID/g in muscle. Similarly, uptake of ^111^In-ADC ranged from 7–10%ID/g in spleen to 0.6–0.9%ID/g in muscle.

At 72 h, little to no effects of predosing were detected in any tissue (including tumor) using non-residualizing ^125^I as a probe for anti-TENB2 uptake (Figure 4C). In contrast, residualizing ^111^In showed predose dependent uptake of the ADC in tumor but not in any other tissue (Figure 4D). Tumor uptakes of ^125^I-ADC at 72 h were relatively flat at 3.4 ± 0.8, 3.7 ± 0.5, 3.6 ± 0.4, and 2.9 ± 0.5 with 0, 0.5, 1, and 3 mg/kg anti-TENB2 predoses, respectively, and 3.8 ± 1.0%ID/g with a 3 mg/kg anti-STEAP1 predose. In contrast, tumor uptakes of ^111^In-ADC at 72 h were predose dependent at 27 ± 6, 28 ± 3, 24 ± 4, and 16 ± 2 with 0, 0.5, 1, and 3 mg/kg anti-TENB2 predoses, respectively, and 35 ± 6%ID/g with a 3 mg/kg anti-STEAP1 predose. Uptake of ^125^I-ADC in other tissues showed no dose dependence and ranged from 3–4%ID/g in spleen to 0.6–0.8%ID/g in muscle. Similarly, uptake of ^111^In-ADC ranged from 4–6%ID/g in spleen to 0.7–0.9%ID/g in muscle.

**Figure 5:**
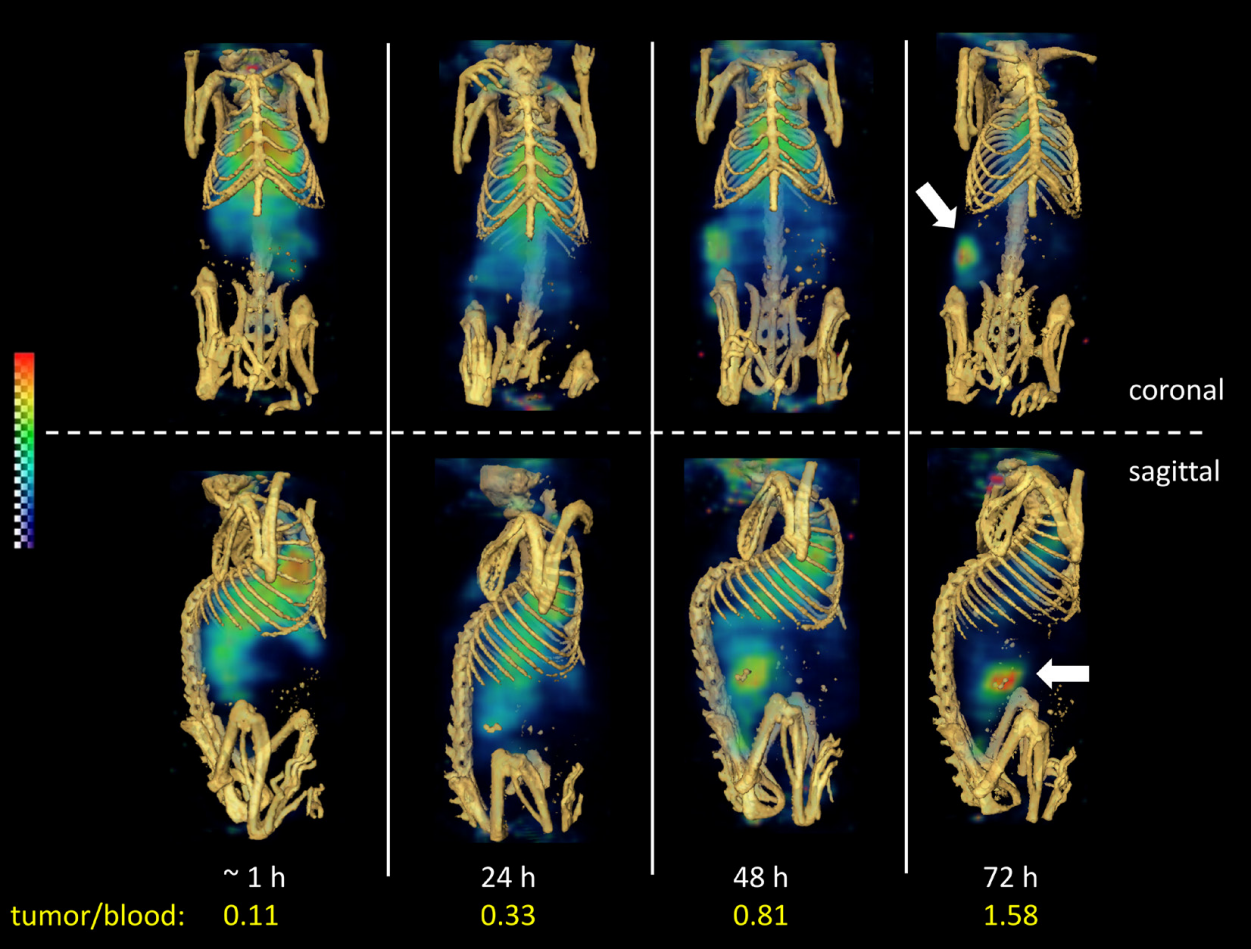
Reconstructed three-dimensional volume rendered SPECT-CT fusion images of ^111^In-ADC (without predose) administered to LuCaP96.1 tumor explant bearing mice intravenously. Both coronal at (*top*) and sagittal (*bottom*) views are included at 1, 24, 48, and 72 hours post-injection.

Non-invasive SPECT-CT imaging of a representative LuCaP96.1 explant bearing mouse receiving ^111^In-ADC is shown in Figure 5. This method permits longitudinal imaging of a single mouse over 4 consecutive days, so that the kinetics of tumor uptake may be visualized. Poor tumor delineation was observed in the SPECT images acquired at 1 and 24 hours (Figure 5), consistent with the 24-hour biodistribution data in Figure 4B in which tumor-to-blood ratios were less than 1. In contrast, at 48 and 72 hours, moderate tumor delineation was achieved (Figure 5), consistent with the 72-hour biodistribution data in Figure 4D, in which tumor uptake was roughly 2-fold higher than blood levels. Tumor/blood ratios derived by SPECT imaging were 0.11, 0.33, 0.81, and 1.58 at 1, 24, 48, and 72 hours, respectively. Overall, these results are consistent with a low (1+) TENB2 expressing tumor model, in striking contrast to our previous imaging data in the high (3+) expressing LuCaP77 model, in which extremely high tumor uptake was observed even at 24 hours post-administration [20].

### ADC tumor growth inhibition study

**Figure 6:**
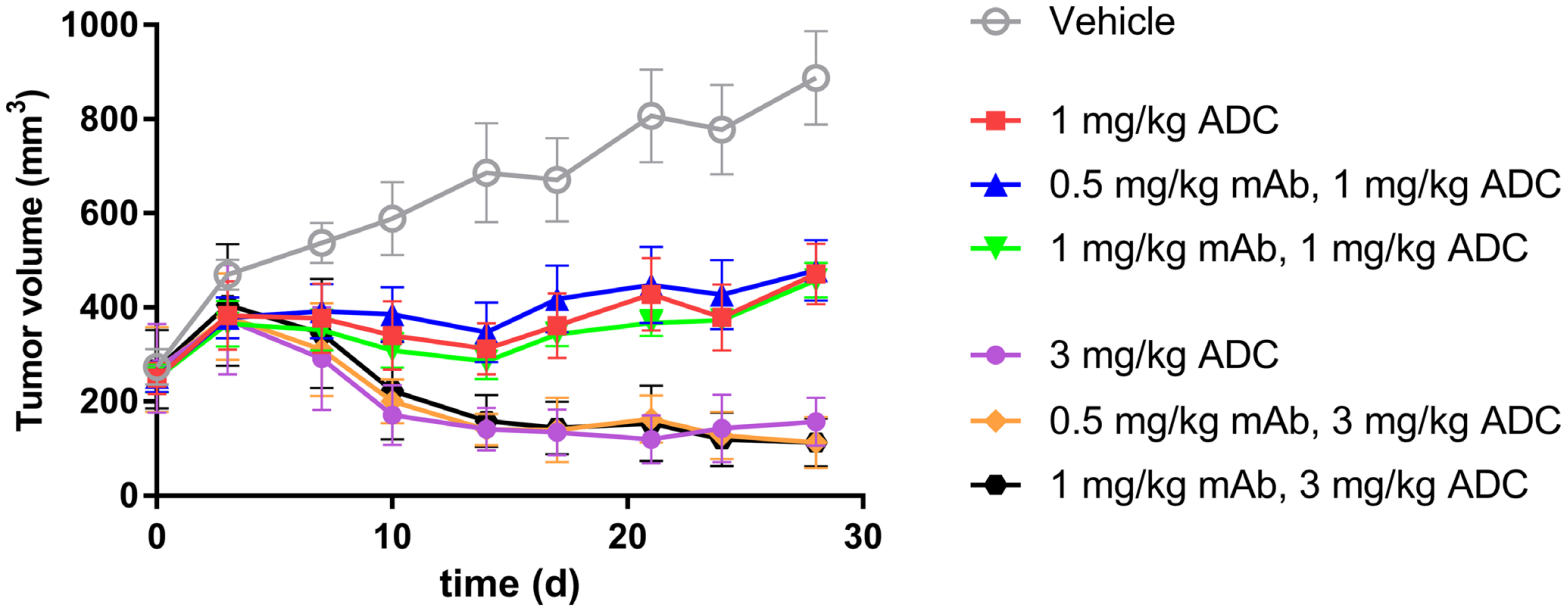
*In vivo* LuCaP96.1 tumor inhibition with anti-TENB2 ADC administered at 1 or 3 mg/kg intravenously in a single dose to male SCID-beige mice bearing established LuCaP96.1 tumor explants. A predose of anti-TENB2 mAb (0, 0.5, or 1 mg/kg) was administered to selected groups at 24 h prior to ADC. In comparison with vehicle control (grey), ADC effectively inhibited LuCaP96.1 tumor growth. The mean (± SEM) tumor volume of all groups versus time is depicted.

To further evaluate the impact of ADC uptake on tumor growth inhibition, we compared the efficacy of ADC in TENB2 expressing LuCaP96.1 model with and without predosing with unconjugated antibody. Predose levels of 0.5 and 1 mg/kg were selected to block low level TENB2 expression in murine intestine, and two dose levels of ADC (1 and 3 mg/kg) were selected to explore the dose dependency of the potential impact from predosing. Tumor growth inhibition was better at 3 mg/kg than at 1 mg/kg (Figure 6), demonstrating that our ADC has dose-dependent efficacy. Comparable tumor inhibitions were observed with or without predosing at 0, 0.5, or 1 mg/kg (Figure 6), suggesting that the predose of anti-TENB2 prior to ADC did not compromise or improve the overall efficacy.

**Figure 7:**
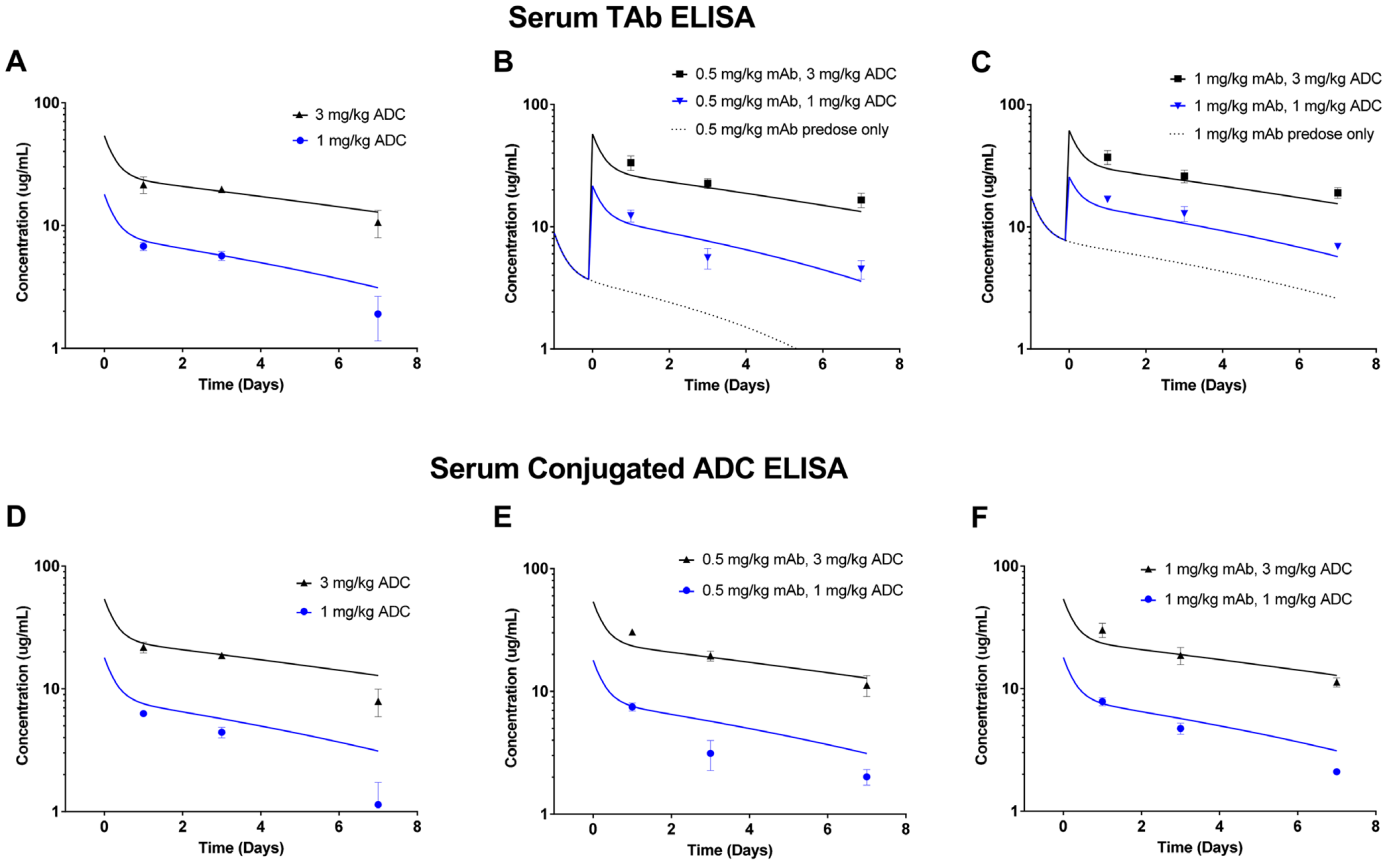
Serum exposure data from mice in the *in vivo* LuCaP96.1 tumor growth inhibition with anti-TENB2 ADC. Overall, observed data points agree well with model simulated PK curves. Serum total antibody ELISA (detecting both mAb and ADC) data for mAb/ADC at predose levels of 0, 0.5 and 1 mg/kg are shown in (**A**), (**B**), and (**C**), respectively. Serum conjugated ADC ELISA (detecting only ADC) data for ADC at mAb predose levels of 0, 0.5, and 1 mg/kg are shown in (**D**), (**E**), and (**F**), respectively. Note that, in (B) and (C), simulated PK curves take into account the contribution of total antibody from both mAb predose and ADC.

We also compared the systemic concentrations of ADC between various dosing groups in the efficacy study. Exposure was analyzed in terms of serum total antibody ELISA (Figure 7A–7C) and serum conjugated ADC ELISA (Figure 7D–7F). Importantly, the total antibody ELISA can detect both the predosed mAb and the ADC, whereas the serum conjugated ADC ELISA detects only ADC. For the total antibody ELISA data, the simulated exposure of the predosed mAb alone is depicted at 0.5 mg/kg (Figure 7B) and at 1 mg/kg (Figure 7C). Overall, the observed mAb/ADC concentrations in serum from mice in the efficacy study agreed well with model simulations of the ADCs alone (Figure 7A, 7D–7F) or in combination with predosed mAb (Figure 7B–7C). Although predosing did not produce dramatic effects on exposure, the observed concentrations did appear to fall closer to simulated curves in the presence of 1 mg/kg predose (Figure 7F) than with no predose (Figure 7D).

## DISCUSSION

The development of novel ADC therapies represents a promising strategy in the treatment of prostate cancer [24]. However, target expression in normal tissue continues to be challenging for the clinical development of ADCs. We previously tested the hypothesis that predosing of unconjugated anti-TENB2 mAb at an optimal dose will saturate specific binding sites for the antibody in normal tissue sinks while retaining sufficient tumor uptake [20]. This approach relies upon a similar biodistribution, but very different toxicity profiles between mAb and ADC.

It is assumed that the level of predose must be fine-tuned in order to avoid saturating tumor receptors. However, it is also plausible that a predose may have a differential ability to block low level antigen expression in a highly perfused tissue sink, while leaving the majority of antigens in a less readily accessible solid tumor microenvironment available for subsequent ADC therapy. Various impediments to the delivery of antibodies to solid tumors have been widely discussed and studied, especially in the context of microspatial distribution [25, 26]. More recent work has suggested that administering a cocktail of mAb and ADC can have better tumor penetration and efficacy than the ADC alone [27]. These data seem to support the original concept of a ‘binding site barrier’ proposed by Weinstein nearly three decades ago [28, 29]. Weinstein postulated that, in cases wherein (i) tumor cells express receptors in very high copy numbers and/or (ii) an antibody binds tumor receptors very tightly, it is plausible that an antibody therapeutic may have limited spatial penetration throughout a tumor due to its ‘consumption’ by the first few layers of tumor cells proximal to the blood vessel from which it extravasated. It is possible that such a binding site barrier could explain the anomalously poor efficacious response of LuCaP77 (3+) to ADC therapy despite high target expression. In contrast, the LuCaP96.1 (1+) might have better response to ADC therapy since the ADC would achieve better penetration throughout the tumor allowing the cytotoxic payload to reach a greater number of overall cells due to lower expression levels. However, besides target expression, differences in other factors like multi-drug resistance [9], sensitivity to MMAE, levels of antigen shedding, and/or tumor explant vascularization could also contribute to these observations. Although our results do not preclude any of the above mechanisms, the much lower tumor uptake of radiolabeled ADC in LuCaP96.1 (~ 30%ID/g at 72 h), relative to previous results in LuCaP77 (> 300%ID/g at 72 h), confirm that the superior efficacy in the 1+ expressing explant model cannot be explained by superior uptake on a whole tumor basis. We have no experimental evidence that significant levels of antigen shedding occur for either of these patient-derived explant models. Furthermore, we confirmed that the considerable tumor uptake of radiolabeled ADC observed in both LuCap77 and LuCap96.1 models was roughly proportional to antigen expression level, suggesting that antigen shedding likely does not play a major role in ADC disposition. However, it is also possible that differences in tumor vascularization and/or interstitial fluid composition can explain the differential efficacy given that a non-binding control antibody (anti-gD) showed modestly (~40%) higher uptake in LuCaP77 than in LuCaP96.1 (8.6 ± 0.5 and 6.2 ± 0.1%ID/g, respectively), suggesting a higher vascular and/or free interstitial pool for the LuCaP77 tumor model [9].

Unlike the MMAE drug payload, our radioactive metal-chelate probe, ^11^1In-DOTA, is residualizing, *i.e.*, ^111^In-DOTA catabolites tend to get entrapped within cells (possibly within lysosomes to an extent) following internalization and degradation of the antibody to which it was originally conjugated [30, 31]. Recent efforts have examined the colocalization (or lack thereof) of ^3^H-labeled MMAE versus the ^111^In-DTPA-labeled antibody for a dual labeled ADC in tumor-bearing mice [32]. The two signals diverged as time progressed, suggesting evidence of the ‘bystander effect’ wherein a given hydrophobic MMAE molecule is able to diffuse across cell membranes beyond the cell in which it was originally released. Nevertheless, agreement between ^3^H (drug) and ^111^In (intact + catabolized antibody) signals at early time points suggest that the radiometal (^111^In) signal is a suitable surrogate for initial ADC deposition. Along these lines, immunoPET signals of another residualizing radiometal probe (^89^Zr) have been previously correlated with efficacy of MMAE-containing ADCs [9]. It is also critical to note that expressing tumor uptake in dose-normalized units (i.e.%ID/g) provides an equivalency between radioactive signal and protein/ADC concentration, such that absolute drug concentrations can easily be calculated as a function of %ID/g tumor uptake, drug-to-antibody ratio and ADC dose [33].

The lack of intestinal uptake observed in the absence of predosing may be rationalized by differences in TENB2 receptor occupancy in the present study conducted at tumor efficacy-relevant doses (≥ 1 mg/kg), in contrast with our previous studies at tracer doses (≤ 0.1 mg/kg) aimed at characterizing the low level antigen sink in intestine (Figure 1). However, the complete absence of evidence of TMDD for our ADC at 1 mg/kg in the present study was somewhat at odds with our previous observations in LuCap77 (3+) tumor bearing mice, for which a 1 mg/kg predose of mAb did not prevent rapid target mediated clearance of a tracer (& 0.1 mg/kg) dose of ADC in the same three-day time period [20]. However, target-mediated clearance of radiolabeled ADC was not observed in normal mice during the first three days after a tracer (& 0.1 mg/kg) dose of radiolabeled ADC was co-administered with 1 mg/kg of non-radiolabeled anti-TENB2 [21]. We conclude that lower levels of TMDD in the LuCaP96.1 (1+) explant model relative to the previous LuCaP77 (3+) is a reasonable explanation for the predose having no effect on the short term (three day) exposure of our ADC at 1 mg/kg in the present study.

Tumor uptake was sensitive to predosing when assayed by ^111^In-DOTA because it is residualizing, *i.e.*, ^111^In-DOTA catabolites tend to get entrapped within cells (possibly also within lysosomes) following internalization and degradation of the antibody to which it was originally conjugated [30, 31]. In contrast, only the levels of intact protein can be detected for antibodies labeled by radioiodination through tyrosine residues with the non-residualizing probe, ^125^I, leading to relatively flat values across predose levels. This discrepancy suggests that receptor engagement and subsequent internalization/catabolism are affected by predosing to a greater extent than the absolute levels of (mostly unbound) intact antibody.

Dose escalation of the anti-TENB2 predose had a dose-dependent effect on ^111^In-ADC uptake in tumor, with competitive inhibition of binding resulting in lower uptake of radiolabeled ADC as the predosed mAb is increased, particularly at 3 mg/kg (Figure 4D). These data suggest that at doses 3 mg/kg and higher, maximal receptor occupancy is being approached in the LuCaP96.1 (1+) explant model. While a 3 mg/kg predose of mAb also reduced tumor uptake of radiolabeled ADC in our previous studies in the LuCaP77 (3+) explant model, even higher amounts of mAb were necessary to fully block tumor uptake, consistent with the vast difference in TENB2 expression levels between these two models. The SPECT-CT imaging data in Figure 5 further support these observations in that only moderate tumor delineation was achieved, with good tumor-to-background signal not evident until 48–72 hours post-injection. Predosing with the anti-STEAP1 control mAb demonstrates that the competition is indeed TENB2 specific, as opposed to being driven by Fc-mediated interactions [34].

To evaluate the impact of predosing and enhanced tumor uptake on efficacy, and to probe the delicate balance between blocking tissue uptake vs. displacing ADC at tumor site, we compared the tumor growth inhibition with or without predosing. ADC administration with predosing at 0.5 and 1 mg/kg preserved (but did not improve) efficacy at two dose levels (Figure 6). ADC doses of 1 and 3 mg/kg were selected based on their respective approximations to IC50 and IC90 values.

In conclusion, this work has extended proof-of-concept for predosing as a strategy for ADC therapy from high expressing (3+) to lower expressing (1+) tumor models. Our work provides evidence that efficacy can be maintained in ADC therapy, even following a predose of unconjugated antibody, to block undesirable uptake in nonmalignant tissues. Predosing approaches could provide a viable alternative to conventional ADC dosing strategies and hold the potential for improved safety profiles for ADC therapeutic regimens.

## MATERIALS AND METHODS

### PK modeling

An integrated, multi-purpose user-friendly GUI application (gPKPDSim) [23] was used to estimate PK parameters from our previously published PK data of ADC in normal mice [21] using a two-compartment model with non-linear clearance (Vm, Km) as previously described [23]. The ODE15s algorithm was used to solve the differential equations, with the data weighted by a proportional error model as previously described (Hosseini et al., 2018). These parameters were used to simulate the dosing regimen use in the LuCaP96.1 explant bearing mice studies. The model-predicted PK profiles were used for comparison with our observed data in those studies. These simulated PK profiles are particularly helpful in delineating circulating total antibody arising from predosed mAb versus ADC.

### Reagents

For biodistribution and tumor growth inhibition studies, a ThioMab against TENB2 (Pr1, affinity 2.3 nM, comparable binding to mTENB2 and hTENB2) was conjugated via two site-specific thiol residues with the auristatin moiety MC-vc-PAB-MMAE, as previously described [11]. The average drug-to-mAb ratio was 1.8–2.0 MMAE per mAb by liquid chromatography-mass spectrometry (LC-MS) analysis. Toxin conjugation via the engineered thiols was previously shown not to significantly alter their antigen binding or PK [35]. Cysteine residues were engineered at Ala114 positions of the heavy chains to produce the ThioMab variant of anti-TENB2 [10]. The ADC was labeled with 1,4,7,10-tetraazacyclododecane-*N*,*N*’,*N*’’,*N*’’’-tetraacetic acid (DOTA) for indium-111 complexation by random modification of lysine residues, as previously described [35]. A separate portion of ADC was subjected to radioiodination by the indirect iodination method, as previously described [21]. Size exclusion HPLC demonstrated > 95% radiochemical purity for all radioimmunoconjugates. Unconjugated anti-TENB2 ThioMab was used for predosing. An anti-six-transmembrane epithelial antigen of prostate 1 (STEAP1, MSTP2109A; affinity 2.4 nM) ThioMab was also constructed as an IgG1 control antibody.

### Patient-derived xenograft models

All animal studies were conducted in accordance with the guidelines of the Institutional Animal Care and Use Committee at Genentech, Inc. Patient-derived human prostate cancer tissues (LuCaP96.1) were kindly provided by Dr. R. Vessella (University of Washington). LuCaP96.1 was derived from prostate tissue [22, 36]. The patient-derived tumors have varying expression of TENB2 and STEAP1 as determined *ex vivo* [9]. The LuCaP96.1 patient derived xenografts were established through serial subcutaneous implantations of 20–30 mm^3^ tumor fragments in intact male CB17 SCID-beige mice.

### ADC biodistribution/PK and imaging study

All *in vivo* protocols, housing, and anesthesia were approved by the Institutional Animal Care and Use Committees of Genentech Laboratory Animal Resources, in compliance with the Association for Assessment and Accreditation of Laboratory Animal Care regulations. The cut-and-count tumor tracer uptake study was conducted in mice bearing LuCaP96.1 patient-derived xenografts (*n* = 3 per group). Male SCID beige mice at 13 weeks old and weighing 25–30 g were implanted subcutaneously with LuCaP96.1 prostate tumor explants, which reached 127–476 mm^3^ by the time of dosing (~ 30 days post-transplant). Dosing solutions were prepared by mixing appropriate amounts of ^111^In-ADC, ^125^I-ADC, and unlabeled ADC. All mice received an intravenous injection of a single dose of ^111^In-ADC (0.185 MBq) mixed with ^125^I-ADC (0.185 MBq), and additional unmodified ADC to deliver a total protein dose of 1 mg/kg. At 24 hours prior to radiotracer, selected groups of mice received a predose of 0.5, 1, or 3 mg/kg of anti-TENB2 mAb, or 3 mg/kg of anti-STEAP1 mAb. Single photon emission computed tomography / X-ray computed tomography (SPECT-CT) imaging was performed on selected mice receiving a higher dose (~ 1 MBq) of ^111^In-ADC only (no predose) as an adjunct to the biodistribution study. Under ketamine/xylazine anesthesia, whole blood was collected in lithium heparin tubes at 5 and 30 minutes, 6 and 10 hours, and 1 and 3 days. Tumors, tissues, and blood were harvested for *ex vivo* analysis at 1 and 3 days post-dose (3 mice per group) for analysis to allow for tissue uptake, mAb internalization, and sufficient background clearance, as previously described [37]. Tumors and tissues were harvested, blot-dried, and weighed for gamma counting with background subtraction and decay correction against dosing solution standards.

### ADC tumor growth inhibition study

The LuCaP96.1 patient derived explant model was also used to evaluate the tumor growth inhibition of anti-TENB2-MMAE, which was administered as a single agent and as a single dose with or without a predose of unconjugated anti-TENB2. Two dose levels of the ADC were evaluated: 1 mg/kg (same dose as for biodistribution) and 3 mg/kg. When tumors reached a volume of 250 ± 30 mm^3^ (31 days post-transplant), animals were randomized into groups of 7 mice each, and given intravenous bolus injection of test materials. Mice received histidine buffer as a vehicle control, 1 or 3 mg/kg ADC only, or a predose of 0.5 or 1 mg/kg anti-TENB2 24 h prior to 1 or 3 mg/kg anti-TENB2-MMAE. Tumor volume growth was monitored for up to 28 days. Tumor length (l, the longest dimension) and width (w, perpendicular to the length) were measured by calipers; tumor volume V was approximated as V = (lw^2^)/2. Sparse PK samples of plasma were collected on days 1, 2, and 7 post ADC dose and analyzed for the concentrations of total antibody or conjugated antibody bearing at least one cytotoxic drug, as previously described [20]. Mice were euthanized before tumors became ulcerated or reached the maximum allowable volume (3000 mm^3^).

## Abbreviations

ADC: antibody drug conjugate
mAb: monoclonal antibody
MMAE: monomethyl auristatin E
PK: pharmacokinetics
PD: pharmacodynamics
SPECT-CT: single photon emission computed tomography – X-ray computed tomography
EGF: epidermal growth factor
TMEFF: transmembrane protein with epidermal growth factor (EGF)-like and two follistatin-like domains
TMDD: target mediated drug distribution
GUI: graphical user interface
DOTA: 1,4,7,10-tetraazacyclododecane-*N*,*N*’,*N*’’,*N*’’’-tetraacetic acid
LC-MS: liquid chromatography – mass spectrometry
SCID: severe combined immunodeficient
STEAP1: six-transmembrane epithelial antigen of prostate 1
ELISA: enzyme-linked immunosorbent assay
